# Draft Genome Sequence of *Bacillus clausii* AKU0647, a Strain That Produces Endo-β-*N*-Acetylglucosaminidase A

**DOI:** 10.1128/genomeA.00310-16

**Published:** 2016-05-05

**Authors:** Yujiro Higuchi, Kazuki Mori, Akiko Suyama, Yibo Huang, Kosuke Tashiro, Satoru Kuhara, Kaoru Takegawa

**Affiliations:** Department of Bioscience and Biotechnology, Faculty of Agriculture, Kyushu University, Fukuoka, Japan

## Abstract

To comprehensively identify glycosyl hydrolase genes in the genome of *Bacillus clausii* strain AKU0647, which produces endo-β-*N*-acetylglucosaminidase A (Endo-A), we conducted whole-genome shotgun sequencing. We identified several other putative glycosyl hydrolase genes apart from the Endo-A gene, and report these findings here.

## GENOME ANNOUNCEMENT

Endo-β-*N*-acetylglucosaminidase (ENGase) is an enzyme that hydrolyzes the N,N′-diacetylchitobiose moiety of the asparagine-linked oligosaccharides of glycoproteins. ENGases belong to two glycosyl hydrolase families, 18 (GH18) and 85 (GH85); distinctively, the former does not exhibit transglycosylation activity but the latter does. The GH85 ENGase A (Endo-A) was originally identified in *Arthrobacter protophormiae* AKU0647 when the strain was cultured in a medium containing ovalbumin, and the enzyme has been well characterized (1–5). However, how this microbe degrades such glycoproteins is not well understood. Therefore, to identify the total complement of glycosyl hydrolase genes in the genome of strain AKU0647, we generated a draft genome sequence.

A whole-genome shotgun sequencing strategy was carried out to acquire the data for the draft sequence. Sequencing was conducted using MiSeq (Illumina) to obtain 300 bp paired-end reads. A total of 5.18 Gbp of data were generated from 1.76 × 10^7^ sequencing reads (1,112-fold-coverage). By assembling the sequence data using the program *Platanus* version 1.2.4, 16 contigs were obtained. The length of the longest contig is 1,277,614 bp and the *N*_50_ size is 713 kb. Genome annotation was performed with Glimmer version 3.02b, BLAST, and InterProScan to identify genes and predict their functions. The draft genome of strain AKU0647 is 4.66 Mbp with an average G+C content of 44.3% and contains 5,047 genes. The gene density is 923 bp/gene. The median and mean gene lengths are 744 bp and 808 bp, respectively.

Unexpectedly, the analysis of this draft sequence revealed that the genome of strain AKU0647 exhibits the highest sequence identity to that of *Bacillus clausii*, not that of *A. protophormiae*, with 100% identity of the 16S rRNA gene sequence. Thus, we renamed this sequenced strain as *B. clausii* AKU0647. According to our annotation of the whole-genome sequence, we confirmed the presence of the Endo-A gene and found two genes of ENGase belonging to GH18, which were designated ORF2621, ORF1208, and ORF2421, respectively. ORF1208 is located on contig0001 and both ORF2421 and ORF2621 are on contig0003 (accession numbers BCXJ01000001 and BCXJ01000003, respectively). Intriguingly, we also discovered four other genes encoding putative glycosyl hydrolases near the locus of the Endo-A gene, all of which might function together with Endo-A to hydrolyze oligosaccharides of glycoproteins. In total, we found 69 putative glycosyl hydrolase genes in the genome of *B. clausii* AKU0647. This draft genome sequence analysis will provide a solid basis for elucidating the enzymatic mechanisms of glycoprotein oligosaccharide degradation by *B. clausii* AKU0647.

### Nucleotide sequence accession numbers.

The contig sequences of *Bacillus clausii* strain AKU0647 have been deposited at DDBJ/EMBL/GenBank under the accession numbers BCXJ01000001 to BCXJ01000016.
